# Temperature potentially induced distinctive flavor of mud crab *Scylla paramamosain* mediated by gut microbiota

**DOI:** 10.1038/s41598-020-60685-0

**Published:** 2020-02-28

**Authors:** Lei Tang, Huan Wang, Chunlin Wang, Changkao Mu, Hongling Wei, Hongzhi Yao, Chunyu Ye, Lizhi Chen, Ce Shi

**Affiliations:** 1 School of Marine Science, Ningbo University, Ningbo, 315211 Zhejiang China; 2 Key Laboratory of Applied Marine Biotechnology, Ministry of Education, Ningbo University, Ningbo, 315211 Zhejiang China; 3 Marine and Fishery Bureau, Sanmen County, Zhejiang Province China; 4 Fishery Technology Station, Sanmen County, Zhejiang Province China

**Keywords:** Bacteriology, Animal physiology

## Abstract

Many factors affect the flavor of crabs. However, impact of temperature on flavor has not been reported. Here, we examined *Scylla paramamosain* collected within the main four producing areas in China from north sampling point (NP) and south sampling point (SP), respectively. The contents of flavouring-related substances in hepatopancreas, muscles and gonads were determined by high-performance liquid chromatography (HPLC). Meanwhile, high-throughput sequencing of 16S RNA gene was used to reveal the diversity distribution of gut microbiota at each sample collection point. Comparisons among flavor substances of edible parts, the implied higher temperature in SP may be beneficial to the accumulation of flavor substances in gonads, while lower temperature in NP may be beneficial to the accumulation of flavor substances in muscles and hepatopancreas. The gut microbiota of crabs, was analyzed via 16S rRNA gene sequencing. The results of gut microbiota showed that there were significant differences in the distribution of gut microbiota in NP and SP. The microbiota composition of SP has a high distribution richness and no absolute dominant bacteria, while NP has absolute dominant bacteria and its microbiota richness was lower than SP. The results of redundancy analysis (RDA) showed that there was a significant correlation between temperature and the relative abundance of gut microbiota, and a significant correlation between gut microbiota and the content of flavor substances. This study indicates that temperature may be one of the main factors for the differences of flavor substances between SP and NP, which was most probably mediated by gut microbiota. Further exploration is needed with laboratory experiments in which the environment is more precisely controlled if these views are to be determined.

## Introduction

Mud crab *Scylla paramamosain* is a commercially important crab widely distributed along the coast of southern China and other Indo-Pacific countries^1–3^. Because of its abundance, fast growth, and high market value, this species is important for both fisheries and aquaculture in southern China^4,5^. *S*. *paramamosain* have been targeted for aquaculture throughout the Asian region^6,7^ and provide commercially viable fishery resources for coastal fishing communities due to high prices in domestic and overseas markets^1,8–11^.

*S*. *paramamosain* has received increasing attention for its great taste, abundant nutrients and medicinal uses^5,12^. The price of the crab depends highly on its flavor. Due to the flavor differences, the market price of mud crabs varies greatly, that good quality mud crabs costing up to $20/kg with more than those of lower quality. A full and well-developed phenotype is often the premise of good quality, especially gonads of female (the gonads of male mud crabs are very small and represent a negligible edible part). Well-developed ovaries of crabs ensure a high market price, and so female crabs with developed ovaries (Fig. S1A,C) receive a higher price compared with male crabs (Fig. S1B,D). Similar to *E*. *sinensis*, *S*. *paramamosain* is ready for sale after gonads mature, and the hepatopancreas (Fig. S1E’a,F’c) and gonads (Fig. S1E’b) are the edible parts most favored by consumers. At this time, the gonads develop rapidly and accumulate nutrients^12–14^.

Salty, sweet, sour, and bitter are the basic flavors of food, with umami increasingly recognized as the fifth basic flavor worldwide^15–17^. Umami is a very important flavor characteristic of aquatic products^18–20^. In addition, Nelson *et al*. found two G-protein binding receptors T1R1 and T1R3 on the surface of taste cells, which allow humans to taste 20 l-amino acids, but had no effect on d-amino acids and other substances^21^. The main flavor substances of seafood aquatic products have been widely reported, in which free amino acids and nucleotides are critical flavor substances^22–24^. Of the amino acids, Glu and Asp are umami amino acids. In addition, umami can be produced from the disodium 5′-monophosphate nucleotides, such as IMP, GMP and AMP, there are high content in aquatic products^25^. Synergistic effects can be produced when flavor nucleotides are present together with Glu and Asp to produce a stronger umami flavor^26^. The level of umami flavor is usually presented in terms of equivalent umami concentration (EUC)^27^. Sweetness is another major flavor in aquatic products such as crabs, because many amino acids provide sweetness, including Gly, Ala, Ser, Thr, Met, Pro, Lys, and Cys; moreover, some amino acids such as His, Arg, Leu, and Ile are slightly sweet amino acids with a minor bitter flavor^28,29^. In addition to the flavor substances, organic acids are important compounds that affect the accumulation of flavor substances in aquatic products^30,31^. The taurine content was higher in the *S*. *paramamosain*, especially in the hepatopancreas of crabs^3^. The taurine is an important nutrient and health material in the diet^32,33^.

The main production areas of mud crabs in China are along the southeast coast to the south of the Yangtze River estuary. Zhejiang and Hainan Provinces are the northernmost and southernmost of the main crab farming areas in China, and production of mud crabs in these two regions in 2016 reached 26549 and 16287 t, respectively^34^. The flavor differences of the mud crab from the two areas is also significantly different, experienced trader could easily tell differences by appearance of crab. Nevertheless, study has not explored the differences in substances of flavor and its potential cause of the mud crab cultured in climatic region. In addition, the gut is an important physiological function organ, which is closely related to the health of the host. Studies have shown that gut microbiota is widely involved in the organ development, nutrient metabolism, immune mechanism and disease regulation of crustaceans^35–37^, and the health, dietary habits and habitat of crustaceans are considered to be the key to the formation of symbiotic intestinal bacterial model^29,38^. A recent study reported that gut microbes impacted the quality of *E*. *sinensis*^30^. Therefore, we hypothesized that temperature differences would affect the diversity distribution of gut microbes and dominant species of crabs, thus affecting the quality and flavor of crabs, which might be the most likely way for temperature to affect the quality and flavor of crabs. In this study, we compared the differences in quality and flavor of mud crabs in the north sampling point (NP) and south sampling point (SP) in China. Meanwhile, high-throughput sequencing based on 16S rRNA gene revealed the diversity distribution of gut microbes and was used to explore the potential relationship among the accumulation of flavor substances, gut microbes and temperature.

## Materials and Methods

### Experimental animals and management

**Figure 1:**
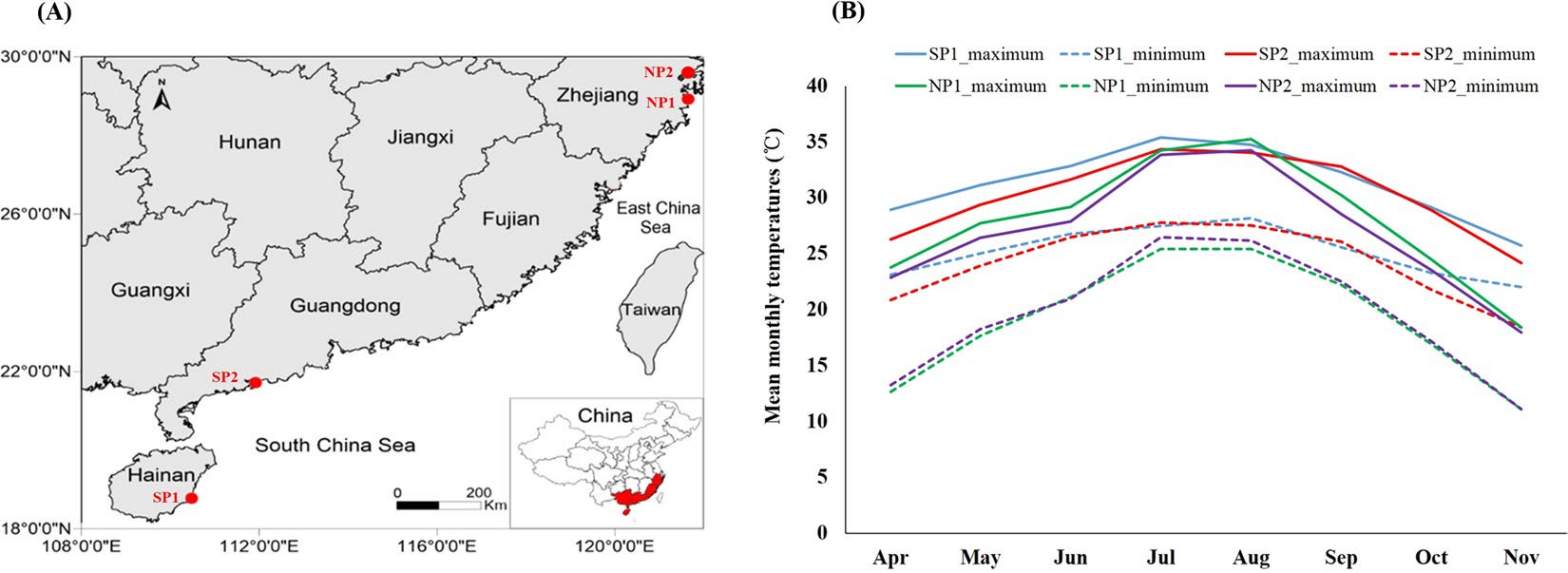
The Northern sampling point (Ninghai and Sanmen) and southern sampling point (Wanning and Yangjiang) of the present experiment (**A**) and temperature change trend from four mud crab breeding areas in 2017. (**B**) SP1 (Wangning county, Hainan province); SP2 (Yangjiang city, Guangdong province); NP1 (Sanmen county, zhejiang province); NP2 (Ninghai county, zhejiang province). “Maximum”indicates the average maximum monthly temperature, and “minimum” indicates the minimum monthly temperature. Solid lines depict the trend of change in average maximum month temperature, and an imaginary line depicts the trend of change in minimum month temperature.

According to the latitude distribution of the main producing areas of mud crabs in China, the southernmost sampling points are Wanning city, Hainan province (SP1) (18 °46′43.29″N, 110 °29′2.89″E) and Yangjiang city, Guangdong province (SP2) (21 °41′53.02″N, 111 °53′21.69″ E), and the northernmost sampling points are Sanmen county, Zhejiang province (NP1) (28 °56′36.32″N, 121 °43′24.30″E) and Ninghai county, Zhejiang province (NP2) (29 °12′11.72″N, 121 °28′30.46″E) (Fig. 1). A total of 7500 seedlings were released in four areas on April 26, 2017, and the initial density was controlled at around 2.5ind/m_2_. Seedlings of juvenile crabs were collected from Zhuhai, Guangdong Province, and mixed cultured with *Penaeus vannamei*, in 3000 m^2^ ponds. The culture protocol including water exchange rate, feeding rate, etc were similar. The crabs in the breeding ponds in all regions use fresh small fish as food. In addition, salinity of each aquaculture water is maintained in the range of 13–16, except for heavy rainfall. Six months later, on 14 November 2017, 9 females and 9 males crabs in the ponds were collected. The sampled crab consistent in weight (300–400 g) was collected. The crabs with good vitality and no damage were captured from the breeding ponds and immediately packaged and transported to the laboratory. Fresh hepatopancreas, muscles and gonads would be taken for experiments immediately after the crabs were killed. The crabs body weight, hepatopancreas index and gonadal index were determined. The results are shown in Fig. S1. The remaining samples were stored at −80 °C. The water quality parameters including temperature, salinity, dissolved oxygen (DO), ammonia-nitroge, nitrite and pH were measured every month throughout the experiment. The values were given in Table S1 as mean ± SD.

### Free amino acids and free nucleotide assays

The edible parts were separated: muscles, hepatopancreas, and gonads of females; or hepatopancreas and muscle of males. The same tissue of three crabs was randomly mixed as one sample, and 3 samples were repeated for each group.

Free amino acids and free nucleotides levels in tissues were tested using high performance liquid chromatography (HPLC), and quantified by external standard methods according to Tao *et al*.^30^. Tissue (1.00 g) of each sample was weighed and mixed with liquid nitrogen for quick grinding, and then added to a centrifuge tube with 1 ml of 5% trichloroacetic acids. The sample was then centrifuged at 10000 *g* for 15 min. The supernatant was filtered through a 0.22-μm filter followed by HPLC testing (Ag1100; NYSE: A, Palo Alto, CA, USA).

### Determination of lactic acid

Lactic acid level was determined spectrophotometrically. The same tissue of three crabs was randomly mixed as one sample, and 3 samples were repeated for each group.Tissue (1.000 g) samples were weighed for each group. Samples were ground in liquid nitrogen followed by addition 1 ml of normal saline. The treated sample was centrifuged at 1000 g for 15 min, and supernatant collected and treated according to the instructions of the lactic acid Assay Kit (Jiancheng, Nanjing, China).

### Equivalent umami concentration (EUC)

The EUC was equivalent umami intensity of mixed umami amino acids and flavor nucleotides compared with a certain concentration of monosodium glutamate (MSG), expressed using the following equation^27^:

$${\rm{EUC}}=\sum \,{{\rm{a}}}_{{\rm{i}}}{{\rm{b}}}_{{\rm{i}}}+1218\,(\sum \,{{\rm{a}}}_{{\rm{i}}}{{\rm{b}}}_{{\rm{i}}})\,(\sum \,{{\rm{a}}}_{{\rm{j}}}{{\rm{b}}}_{{\rm{j}}})$$

where EUC was equivalent MSG (g MSG/100 g), a_i_ was the concentration of flavor amino acids (Asp or Glu) (g/100 g), b_i_ was the relative freshness coefficient of umami amino acids relative to MSG (Glu: 1; and Asp: 0.077), a_j_ was the concentration of flavor nucleotides (5′-IMP, 5′-GMP and 5′-AMP) (g/100 g), b_j_ was the relative conversion coefficient of flavor nucleotides (5′-IMP: 1; 5′-GMP: 2.3; and AMP: 0.18), and 1218 was a synergistic interaction constant.

### DNA extraction and amplification

A total of 9 males and 9 females gut bacterial DNA of crabs was extracted from each collection site sample collection point. Total genomic DNA was extracted using DNA Extraction Kit following the manufacturer’s instructions (DNeasy PowerSoil Kit). Quality and quantity of DNA was verified with NanoDrop and agarose gel. Extracted DNA was diluted to a concentration of 1 ng/μl and stored at −20 °C until further processing.

The diluted DNA was used as a template for PCR amplification of bacterial 16S rRNA genes with the barcoded primers and HiFi Hot Start Ready Mix (KAPA). For bacterial diversity analysis, V3-V4 variable regions of 16S rRNA genes was amplified with universal primers 343 F (5′-TACGGRAGGCAGCAG -3′) and 798 R (5′- AGGGTATCTAATCCT-3′).

### Bioinformatic analysis

Amplicon quality was visualized using gel electrophoresis, purified with AMPure XP beads (Agencourt), and amplified for another round of PCR. After purified with the AMPure XP beads again, the final amplicon was quantified using Qubit dsDNA assay kit. Equal amounts of purified amplicon were pooled for subsequent sequencing.

Before sequencing, 3 samples were randomly selected from 9 samples in each group as one sample, and 3 samples were repeated for each group. The 16S amplicon sequencing was performed on the Illumina MiSeq platform by Shanghai OE Biotech Co., Ltd. Raw sequencing data were in FASTQ format. Paired-end reads were then preprocessed using Trimmomatic software to detect and cut off ambiguous bases. It also cut off low quality sequences with average quality score below 20 using sliding window trimming approach. After trimming, paired-end reads were assembled using FLASH software. Parameters of assembly were: 10 bp of minimal overlapping, 200 bp of maximum overlapping and 20% of maximum mismatch rate. Sequences were performed further denoising as follows: reads with ambiguous, homologous sequences or below 200 bp were abandoned. Reads with 75% of bases above Q20 were retained. Then, reads with chimera were detected and removed. These two steps were achieved using QIIME software (version 1.8.0).

Clean reads were subjected to primer sequences removal and clustering to generate operational taxonomic units (OTUs) using UPARSE software with 97% similarity cutoff. The representative read of each OTU was selected using QIIME package. All representative reads were annotated and blasted against Silva database Version 123 (or Greengens) (16S rDNA) using RDP classifier (confidence threshold was 70%).

### Statistical analysis

The data were analyzed by a SPSS for Windows (Version 21.0) statistical package. Prior to analysis, raw data were diagnosed for normality of distribution and homogeneity of variance with Kolmogorov-Smirnov test, and Levene’s test respectively. Non-normal and heterogeneous data were transformed until normality and homogeneity were achieved. Two-way analysis of variance (ANOVA) was used to compare the effects of treatments on accumulation of taurine and lactic acid, flavor substances followed by Tukey multiple comparison post hoc test. Differences were considered significant at a probability level of 0.05. Redundancy analysis (RDA) was performed to explore the correlations between bacteria at the phylum level of percentage and envionment variables, flavor substances.

### Ethical approval statement

The animal subjects used in the study were crabs, which are invertebrates and exempt from this requirement. All experiments were performed according to the Experimental Animal Management Law of China and approved by the Animal Ethics Committee of Ningbo University.

## Results

### Temperature differences in NP and SP ponds

Due to the differences in geographical locations, temperatures in the SP and NP greatly differ (Fig. 1), but water quality parameters such as salinity, dissolved oxygen (DO), ammonia nitrogen, nitrite and pH value measured every month are very similar, with no significant differences (Table S1). The annual temperature variation range of SP region was smaller than that of NP region. The maximum temperature was about 38 °C and the minimum temperature was about 18 °C. However, the annual temperature variation in the NP region was polytropic. The temperature change curve for April–November was nearly a parabola. Temperature gradually increased during April–July and gradually decreased during August-November. Except for July and August, temperatures at all other times were lower than that of the SP region (Fig. 1). Moreover, upon seedling release on 26 April 2017, the highest temperature in NP was 23 °C and the lowest was 15 °C; and corresponding temperatures in SP were 29 °C and 23 °C. When the crabs were caught on 14 November 2017, the highest temperature in NP was 19 °C and the lowest was 10 °C; the corresponding temperatures in SP were 26 and 22 °C.

### Comparison of flavor substances between NP and SP crabs

**Figure 2:**
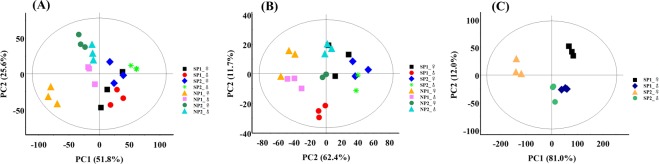
Principal component analysis (PCA) of flavor substances in three edible parts from four sampling sites in north and south China.

The principal component analysis (PCA) of the composition and content of flavor substances (free amino acids and free nucleotides) in three edible parts of the four sample collection points in north and south China was shown in Fig. 2. As can be seen from the score chart of flavor substances in hepatopancreas (Fig. 2A), samples from all regions were within confidence intervals, with relatively concentrated sample distribution and clear boundaries between samples, which were mainly divided into two categories: Samples from two collection points in southern region (SP) were distributed in the positive region to the right of PC1, while samples from two collection points in northern region (NP) were distributed in the negative region to the left of PC1. This indicates that the composition and content of flavor substances of SP1 and SP2 in the southern region are similar, and that of NP1 and NP2 in the northern region are similar, and that the content of flavor substances in the northern and southern regions are significantly differences. PCA scores of muscles (Fig. 2B) and gonads (Fig. 2C) were not as distinct as those of hepatopancreas, but the trends were similar. In addition, according to the distribution of male and female samples in each region, it can be concluded that there are also male and female differences in the accumulation of flavor substances.

**Figure 3:**
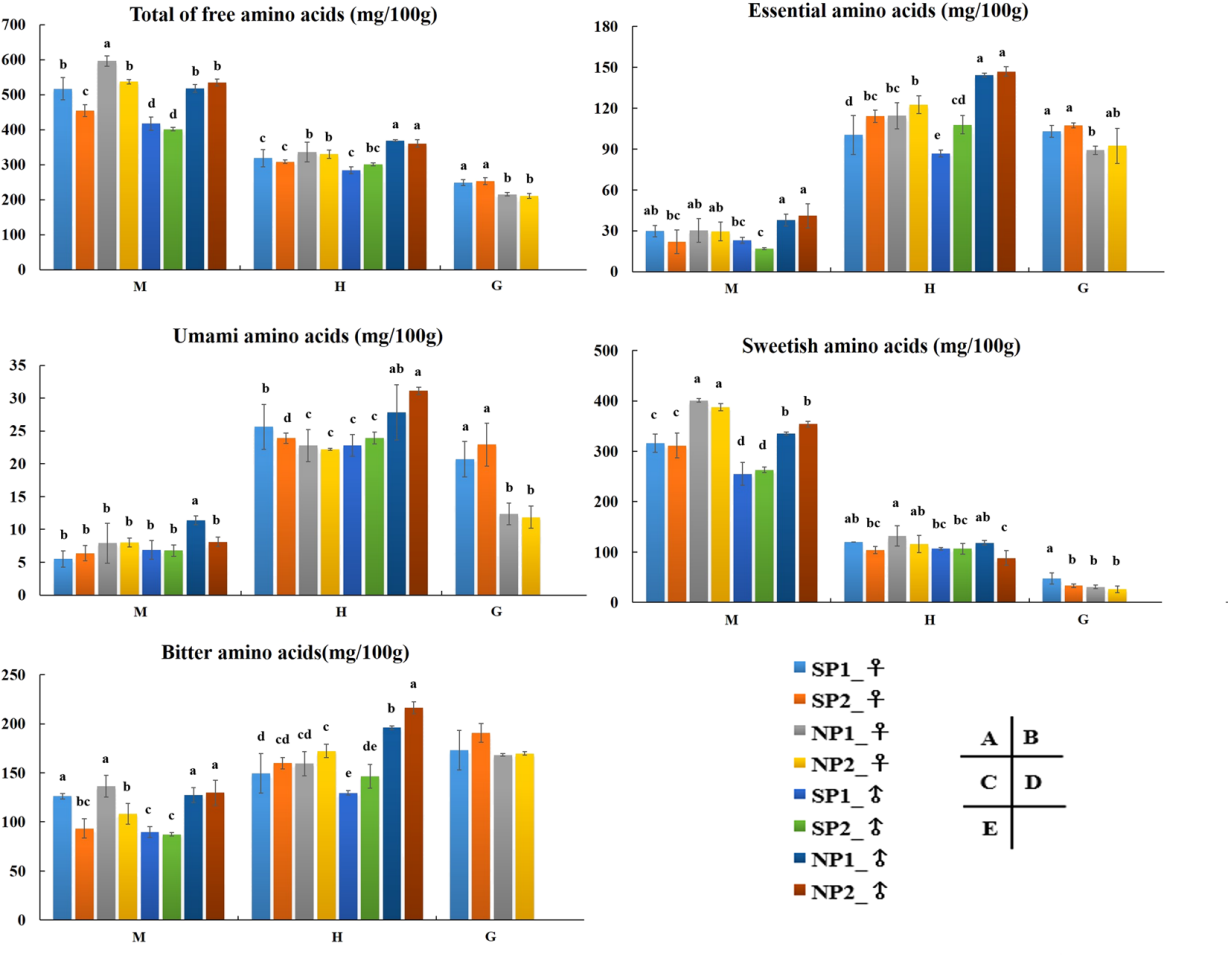
Content of total free amino acids (**A**), Essential amino acids (**B**), Sweetish amino acids (**C**), Umami amino acids (**D**,**E**), bitter amino acids in the three tissues. Essential amino acids include isoleucine, leucine, lysine, methionine, phenylalanine, threonine, tryptophan, and valine. Sweetish amino acids include alanine, glycine, proline, serine, and threonine. Umami amino acids include aspartic acid and glutamic acid. Bitter amino acids include arginine, isoleucine, leucine, methionine, phenylalanine, tyrosine, tryptophan, and valine^28,50^. Different letters above the columns indicate a significant differences (P < 0.05) between SP1-♀, SP2-♀, NP1-♀, NP2-♀, SP1-♂, SP2-♂, NP1-♂ and NP2-♂ respectively in muscle (M), hepatopancreas (H), and gonads (G).

Analyses of free amino acids in three edible parts (muscles, hepatopancreas, and gonads) showed that muscle had the largest protein pool of the crabs, which was the greatest amount of total amino acids (TAA, which here refers in particular to the sum of free amino acids in a tissue) (Fig. 3A). Of TAA, 80% was concentrated in four free amino acids: Pro, Arg, Gly, and Ala (Tables 1–3). Muscles showed the most abundant TAA, followed by hepatopancreas, and gonads. Regarding TAA levels in muscles and hepatopancreas, NP1 and NP2 were significantly higher than those of SP1 and SP2, respectively, and the levels were higher in females with the highest level in NP1-♀ of 596.08 ± 14.97 mg/100 g in musles (Table 1). The gonads were opposite, showed SP1 and SP2 were significantly higher than those of NP1 and NP2 (Fig. 3A and Table 3). The highest essential acids (EAA) level was in hepatopancreas, and far above muscles (Fig. 3B and Tables 1–2). Among the edible parts of female crabs, there was no significant differences in EAA between SP_♀ and NP_♀, but in male crabs, NP_♂ was significantly higher than SP_♂. The accumulation of umami amino acids (UAA) was higher in hepatopancreas and gonads, especially in hepatopancreas. There was no significant differences between SP_♀ and NP_♀ in the content of UAA in hepatopancreas and muscles, but SP_♀ is significantly higher than NP_♀ in the gonads, and there was no significant differences in male crabs (Fig. 3C). Sweetish amino acids (SAA) were far higher in muscle than in hepatopancreas and gonads. There was no significant differences in the content of SAA between SP and NP in hepatopancreas and gonads, but NP was significantly higher than SP in muscles, and there was no differences between male and female. The highest value was for NP1-♀, reaching 401.03 ± 3.57 mg/100 g, and accounted for 67.3% of TAA (Fig. 3D and Table 1). Bitter amino acids (BAA) levels were slightly lower in muscles and higher in hepatopancreas and gonads (Fig. 3E and Tables 1–3). There was no significant differences in BAA content between SP and NP in edible parts of female crabs, but NP content was significantly higher than SP in male crabs.

**Table 1:** Content of free amino acid in muscle of *S*. *paramamosain* from different areas (mg/100 g).

FAA	Taste	Females				Males			
		SP1-♀	SP2-♀	NP1-♀	NP2-♀	SP1-♂	SP2-♂	NP1-♂	NP2-♂
Asp	Umami (+)	0.96 ± 0.28	1.15 ± 0.79	0.46 ± 0.02	0.70 ± 0.22	0.61 ± 0.19	0.50 ± 0.06	0.83 ± 0.64	0.54 ± 0.23
Glu	Umami (+)	4.55 ± 0.96c	5.23 ± 1.25bc	7.43 ± 3.02b	7.32 ± 0.45b	6.29 ± 1.38bc	6.27 ± 0.86bc	10.57 ± 0.22a	7.56 ± 0.94ab
Ser	Sweet (+)	1.06 ± 0.72c	0.67 ± 0.24c	1.99 ± 0.21c	5.22 ± 3.65b	0.95 ± 0.49c	1.18 ± 0.05c	1.37 ± 0.39c	8.90 ± 2.92a
Gly	Sweet (+)	180.67 ± 12.31d	188.89 ± 14.47cd	191.15 ± 13.17cd	246.55 ± 9.70a	160.48 ± 9.73e	185.75 ± 4.83cd	202.65 ± 13.29c	223.43 ± 11.89b
Thr	Sweet (+)	2.67 ± 0.60c	3.08 ± 1.15c	9.15 ± 2.66a	7.62 ± 0.53ab	2.40 ± 0.69c	2.31 ± 0.31c	5.44 ± 1.44b	8.12 ± 1.29a
Ala	Sweet (+)	37.04 ± 4.50bc	32.97 ± 4.24cd	49.00 ± 10.81a	30.03 ± 3.57cd	31.35 ± 2.41cd	23.31 ± 3.49d	43.94 ± 7.30ab	29.32 ± 1.56cd
Pro	Sweet (+)	94.45 ± 22.29b	85.72 ± 15.66b	149.73 ± 8.20a	98.33 ± 3.50b	59.77 ± 17.30c	50.67 ± 4.64c	81.95 ± 6.43b	84.21 ± 1.17b
His	Bitter (−)	1.83 ± 0.04c	1.09 ± 0.23c	4.05 ± 0.96a	4.39 ± 0.46a	1.45 ± 0.16c	1.21 ± 0.04c	3.05 ± 0.40b	4.50 ± 0.44a
Arg	Bitter (−)	94.00 ± 7.70b	71.01 ± 1.51d	108.95 ± 10.68a	79.65 ± 3.95cd	64.72 ± 4.6e	69.55 ± 0.68de	86.88 ± 3.60cd	88.20 ± 4.79bc
Tyr	Bitter (−)	3.12 ± 0.93bc	2.40 ± 0.75c	2.40± 0.68c	2.26 ± 0.26c	3.06 ± 0.66bc	2.02 ± 0.29c	4.84 ± 0.93a	4.10 ± 0.29ab
Val	Bitter (−)	4.48 ± 0.47ab	3.58 ± 0.80b	4.32 ± 1.40ab	3.42 ± 0.85b	4.83 ± 0.60ab	3.46 ± 0.16b	4.82 ± 0.30ab	5.65 ± 1.50a
Met	Bitter (−)	2.92 ± 0.55bc	2.08 ± 0.79c	3.39 ± 0.87bc	4.03 ± 0.93b	2.29 ± 0.29c	2.25 ± 0.62c	8.55 ± 0.66a	9.65 ± 0.77a
Trp	Bitter (−)	0.99 ± 0.18b	0.43 ± 0.31c	0.35 ± 0.22c	0.14 ± 0.09c	1.00 ± 0.25b	0.23 ± 0.04c	1.49 ± 0.27a	0.41 ± 0.01c
Phe	Bitter (−)	1.98 ± 0.22ab	1.08 ± 0.57c	0.69 ± 0.16c	1.02 ± 0.22c	2.05 ± 0.34ab	1.16 ± 0.13c	2.56 ± 0.35a	1.83 ± 0.40b
Ile	Bitter (−)	2.96 ± 0.44a	1.50 ± 0.66c	1.31 ± 0.41c	1.35 ± 0.64c	2.23 ± 0.43abc	1.76 ± 0.15bc	2.7 ± 0.38abc	2.17 ± 0.85abc
Leu	Bitter (−)	4.88 ± 0.80abc	2.99 ± 1.20c	3.71 ± 1.25bc	4.51 ± 2.08abc	3.65 ± 0.56bc	2.76 ± 0.16c	5.87 ± 0.80ab	6.46 ± 2.62a
Lys	Bitter (−)	8.97 ± 3.74a	7.20 ± 3.45ab	7.4 ± 2.26a	7.43 ± 1.70a	4.51 ± 0.32ab	2.88 ± 0.30b	6.53 ± 1.37ab	6.74 ± 2.46ab
Asn	tasteless	2.33 ± 0.73b	1.36 ± 0.46cd	2.52 ± 0.50b	2.83 ± 0.55b	2.27 ± 0.22b	2.15 ± 0.29bc	1.02 ± 0.53d	3.77 ± 0.49a
Gln	tasteless	65.92 ± 20.94a	41.36 ± 10.92cd	48.04 ± 1.69bc	29.94 ± 2.70d	62.32 ± 5.03ab	41.67 ± 5.91cd	42.13 ± 2.90cd	39.2 ± 2.65cd
Cys-s	tasteless	1.08 ± 0.02b	0.47 ± 0.11c	0.03 ± 0.02d	0.18 ± 0.12d	1.29 ± 0.07a	0.50± 0.17c	0.59 ± 0.08c	0.01 ± 0.01d
UAA	Umami (+)	5.51 ± 1.23b	6.38 ± 1.17b	7.89 ± 3.04b	8.02 ± 0.66b	6.90 ± 1.42b	6.77 ± 0.87b	11.41 ± 0.66a	8.09 ± 0.71b
SAA	Sweet (+)	315.88 ± 17.81c	311.32 ± 24.85c	401.03 ± 3.57a	387.75 ± 7.11a	254.95 ± 22.47d	263.23 ± 5.42d	335.35 ± 3.03bc	353.97 ± 5.72b
BAA	Bitter (−)	126.13 ± 2.73a	93.36 ± 9.73bc	136.58 ± 11.1a	108.19 ± 10.5b	89.78 ± 5.68c	87.29 ± 1.79c	127.28 ± 7.46a	129.71 ± 12.77a
EAA	—	29.85 ± 4.13ab	21.94 ± 8.65bc	30.33 ± 8.58ab	29.51 ± 6.81ab	22.96 ± 2.18bc	16.81 ± 0.74c	37.95 ± 4.25a	41.03 ± 8.96a
TAA	—	516.86 ± 31.23b	454.25 ± 17.29c	596.08 ± 14.97a	536.9 ± 5.79b	417.5 ± 18.94d	401.6 ± 4.89d	517.78 ± 11.32b	534.76 ± 9.35b

In the same row, different letters means significant difference; (P < 0.05). SP1 (Wangning county, Hainan province); SP2 (Yangjiang city, Guangdong province); NP1 (Sanmen county, zhejiang province); NP2 (Ninghai county, zhejiang province). −, Indicate could not be determined.

**Table 2:** Content of free amino acid in hepatopancreas of *S*. *paramamosain* from different areas (mg/100 g).

FAA	Taste	Females				Males			
		SP1-♀	SP2-♀	NP1-♀	NP2-♀	SP1-♂	SP2-♂	NP1-♂	NP2-♂
Asp	Umami (+)	5.34 ± 1.72bc	4.09 ± 0.38c	4.04 ± 0.75c	4.64 ± 1.15c	3.80 ± 0.73c	3.97 ± 0.59c	6.36 ± 0.94ab	7.75 ± 0.55a
Glu	Umami (+)	20.3 ± 2.21abc	16.83 ± 0.55c	18.76 ± 2.13bc	17.53 ± 1.28c	19.00 ± 1.92bc	19.98 ± 0.78abc	21.49 ± 3.29ab	23.35 ± 0.30a
Ser	Sweet (+)	9.73 ± 1.10b	9.87 ± 0.68b	12.01 ± 0.70a	9.79 ± 0.24b	7.05 ± 0.34c	10.16 ± 0.99b	12.35 ± 0.37a	7.37 ± 0.47c
Gly	Sweet (+)	43.31 ± 11.69ab	32.79 ± 7.04bc	41.97 ± 3.37ab	39.89 ± 8.44ab	48.84 ± 5.04a	41.45 ± 10.31ab	47.50 ± 4.40a	23.83 ± 2.68c
Thr	Sweet (+)	8.78 ± 1.60c	8.76 ± 0.53c	12.83 ± 1.20ab	11.62 ± 0.48b	6.19 ± 0.58d	9.40 ± 1.02c	11.76 ± 0.94b	13.62 ± 0.04a
Ala	Sweet (+)	23.99 ± 2.34a	16.93 ± 2.36b	21.73 ± 0.83ab	21.70 ± 1.38ab	21.56 ± 6.68ab	20.99 ± 2.22ab	20.12 ± 1.41ab	20.42 ± 1.78ab
Pro	Sweet (+)	34.30 ± 8.42ab	35.39 ± 2.97ab	43.65 ± 19.22b	32.90 ± 7.18ab	22.90 ± 2.20a	24.73 ± 3.39a	26.64 ± 4.02ab	23.02 ± 10.35a
His	Bitter (−)	3.45 ± 0.72cd	3.69 ± 0.14c	5.10 ± 0.41b	5.35 ± 0.18b	2.80 ± 0.17d	3.25 ± 0.62cd	6.21 ± 0.37a	6.74 ± 0.06a
Arg	Bitter (−)	41.49 ± 4.30ab	35.39 ± 4.41bcd	39.77 ± 2.84abc	41.90 ± 2.74a	34.11 ± 3.15cd	31.16 ± 5.17d	40.67 ± 1.00ab	45.62 ± 1.39a
Tyr	Bitter (−)	13.01 ± 2.49b	15.46 ± 1.61b	12.87 ± 1.14b	14.01 ± 0.79b	12.14 ± 2.29b	13.67 ± 0.28b	16.61 ± 0.89b	30.74 ± 9.43a
Val	Bitter (−)	9.91 ± 1.94b	11.41 ± 0.78bc	12.46 ± 0.99b	11.50 ± 0.56bc	8.18 ± 1.16d	11.08 ± 0.48bc	15.38 ± 0.69a	15.23 ± 0.42a
Met	Bitter (−)	8.64 ± 1.53c	9.60 ± 0.38c	8.89 ± 0.63c	10.91 ± 0.72b	7.28 ± 0.35d	9.26 ± 0.51c	12.83 ± 0.89a	13.44 ± 0.12a
Trp	Bitter (−)	4.90 ± 0.88b	4.30 ± 0.68bc	3.71 ± 0.39c	3.74 ± 0.73c	4.51 ± 0.44bc	3.75 ± 0.27c	6.72 ± 0.40a	7.56 ± 0.23a
Phe	Bitter (−)	11.79 ± 2.75bc	14.01 ± 0.85b	12.13 ± 1.3bc	14.12 ± 1.56b	10.21 ± 1.19c	12.56 ± 1.09bc	19.03 ± 0.50a	19.77 ± 0.20a
Ile	Bitter (−)	7.50 ± 1.87cd	9.26 ± 0.46b	10.00 ± 1.01b	10.06 ± 0.80b	6.53 ± 1.01d	8.51 ± 0.49bc	12.54 ± 0.15a	12.31 ± 0.74a
Leu	Bitter (−)	25.34 ± 4.14de	30.69 ± 1.99	28.15 ± 2.18cde	34.48 ± 2.07ab	24.25 ± 0.75e	29.26 ± 2.90cd	38.32 ± 0.90a	37.04 ± 0.50a
Lys	Bitter (−)	23.51 ± 3.71ab	26.06 ± 2.53a	26.34 ± 2.27a	26.16 ± 0.81a	19.59 ± 1.50b	24.06 ± 3.15a	27.71 ± 1.47a	27.95 ± 2.63a
Asn	tasteless	4.36 ± 0.81bc	4.45 ± 0.14bc	5.26 ± 0.55b	4.43 ± 0.40bc	3.60 ± 0.15c	4.66 ± 0.57b	6.60 ± 0.69a	7.28 ± 0.21a
Gln	tasteless	15.81 ± 0.51	15.95 ± 3.73	13.56 ± 1.29	13.64 ± 0.46	19.00 ± 10.10	16.36 ± 2.86	17.26 ± 1.98	14.87 ± 1.88
Cys-s	tasteless	3.17 ± 1.19ab	3.48 ± 0.33a	2.40 ± 0.17bc	1.75 ± 0.19c	2.34 ± 0.32bc	2.51 ± 0.04bc	2.87 ± 0.04ab	2.76 ± 0.09ab
UAA	Umami (+)	25.64 ± 3.41bc	20.92 ± 0.78d	22.80 ± 2.44cd	22.17 ± 0.15cd	22.79 ± 1.64cd	23.94 ± 0.88bcd	27.85 ± 4.20ab	31.10 ± 0.57a
SAA	Sweet (+)	120.11 ± 0.23ab	103.75 ± 6.99bc	132.19 ± 19.88a	115.91 ± 17.15ab	106.54 ± 2.15bc	106.73 ± 10.46bc	118.38 ± 4.73ab	88.27 ± 14.33c
BAA	Bitter (−)	149.54 ± 20.19d	159.87 ± 5.66cd	159.42 ± 12.41cd	172.24 ± 6.76c	129.60 ± 2.12e	146.57 ± 12.16de	196.02 ± 1.85b	216.41 ± 5.99a
EAA	—	100.37 ± 14.29d	114.09 ± 4.49bc	114.52 ± 9.59bc	122.60 ± 6.63b	86.75 ± 2.63e	107.87 ± 6.60cd	144.3 ± 1.55a	146.93 ± 3.49a
TAA	—	318.63 ± 24.79cd	308.41 ± 4.89cde	335.64 ± 28.12bc	330.14 ± 12.36c	283.88 ± 9.45e	300.77 ± 3.91de	368.98 ± 2.41a	360.69 ± 10.29ab

In the same row, different letters means significant difference; (P < 0.05). SP1 (Wangning county, Hainan province); SP2 (Yangjiang city, Guangdong province); NP1 (Sanmen county, zhejiang province); NP2 (Ninghai county, zhejiang province). −, Indicate could not be determined.

**Table 3:** Content of free amino acid in gonad of *S*. *paramamosain* from different areas (mg/100 g).

FAA	Taste	Females			
		SP1-♀	SP2-♀	NP1-♀	NP2-♀
Asp	Umami (+)	2.10 ± 0.16b	4.21 ± 1.72a	0.90 ± 0.09b	0.84 ± 0.14b
Glu	Umami (+)	18.61 ± 2.53a	18.71 ± 2.04a	11.45 ± 1.72b	11.03 ± 1.55b
Ser	Sweet (+)	0.05 ± 0.02	0.03 ± 0.01	0.06 ± 0.01	0.13 ± 0.13
Gly	Sweet (+)	8.69 ± 2.52	6.08 ± 1.91	6.42 ± 0.47	7.24 ± 1.64
Thr	Sweet (+)	2.35 ± 0.45	2.58 ± 0.53	2.65 ± 0.38	2.53 ± 0.93
Ala	Sweet (+)	15.95 ± 2.55a	8.93 ± 2.41b	6.16 ± 1.10b	6.27 ± 0.59b
Pro	Sweet (+)	20.77 ± 9.53	15.65 ± 4.20	15.62 ± 2.23	10.12 ± 3.44
His	Bitter (−)	3.07 ± 0.42ab	1.83 ± 0.31b	2.87 ± 0.69ab	3.67 ± 0.95a
Arg	Bitter (−)	57.62 ± 18.13	72.55 ± 7.26	71.23 ± 4.71	69.35 ± 14.70
Tyr	Bitter (−)	11.6 ± 1.62a	11.63 ± 3.37a	7.77 ± 1.10ab	6.83 ± 1.37ab
Val	Bitter (−)	2.99 ± 0.49	2.48 ± 1.06	1.96 ± 0.13	1.90 ± 0.60
Met	Bitter (−)	1.49 ± 0.08b	1.89 ± 0.15a	1.29 ± 0.10b	1.24 ± 0.33b
Trp	Bitter (−)	63.43 ± 7.46ab	68.86 ± 3.44a	51.71 ± 2.64b	52.91 ± 10.31b
Phe	Bitter (−)	2.51 ± 0.49	2.50 ± 0.46	1.98 ± 0.37	2.71 ± 0.86
Ile	Bitter (−)	1.47 ± 0.22	1.25 ± 0.34	0.96 ± 0.07	1.14 ± 0.40
Leu	Bitter (−)	2.32 ± 0.61	2.27 ± 1.27	1.54 ± 0.28	2.13 ± 1.09
Lys	Bitter (−)	26.46 ± 7.59	25.55 ± 1.75	27.05 ± 4.39	27.91 ± 1.95
Asn	tasteless	1.39 ± 0.40a	0.65 ± 0.01b	0.83 ± 0.03b	0.85 ± 0.15b
Gln	tasteless	6.02 ± 0.69a	5.25 ± 1.85ab	3.37 ± 0.59bc	2.13 ± 0.07c
Cys-s	tasteless	0.08 ± 0.02	0.07 ± 0.02	0.04 ± 0.01	0.06 ± 0.03
UAA	Umami (+)	20.72 ± 2.69a	22.92 ± 3.29a	12.35 ± 1.64b	11.87 ± 1.67b
SAA	Sweet (+)	47.81 ± 10.85a	33.27 ± 3.40b	30.91 ± 3.20b	26.29 ± 6.66b
BAA	Bitter (−)	172.95 ± 20.17	190.81 ± 9.57	168.35 ± 1.39	169.77 ± 1.85
EAA	—	103.01 ± 4.47ab	107.38 ± 1.85a	89.13 ± 3.11c	92.46 ± 12.80bc
TAA	—	248.96 ± 8.02a	252.98 ± 9.62a	215.85 ± 5.38b	210.97 ± 7.13b

In the same row, different letters means significant difference; (P < 0.05). SP1 (Wangning county, Hainan province); SP2 (Yangjiang city, Guangdong province); NP1 (Sanmen county, zhejiang province); NP2 (Ninghai county, zhejiang province). −, Indicate could not be determined.

**Figure 4:**
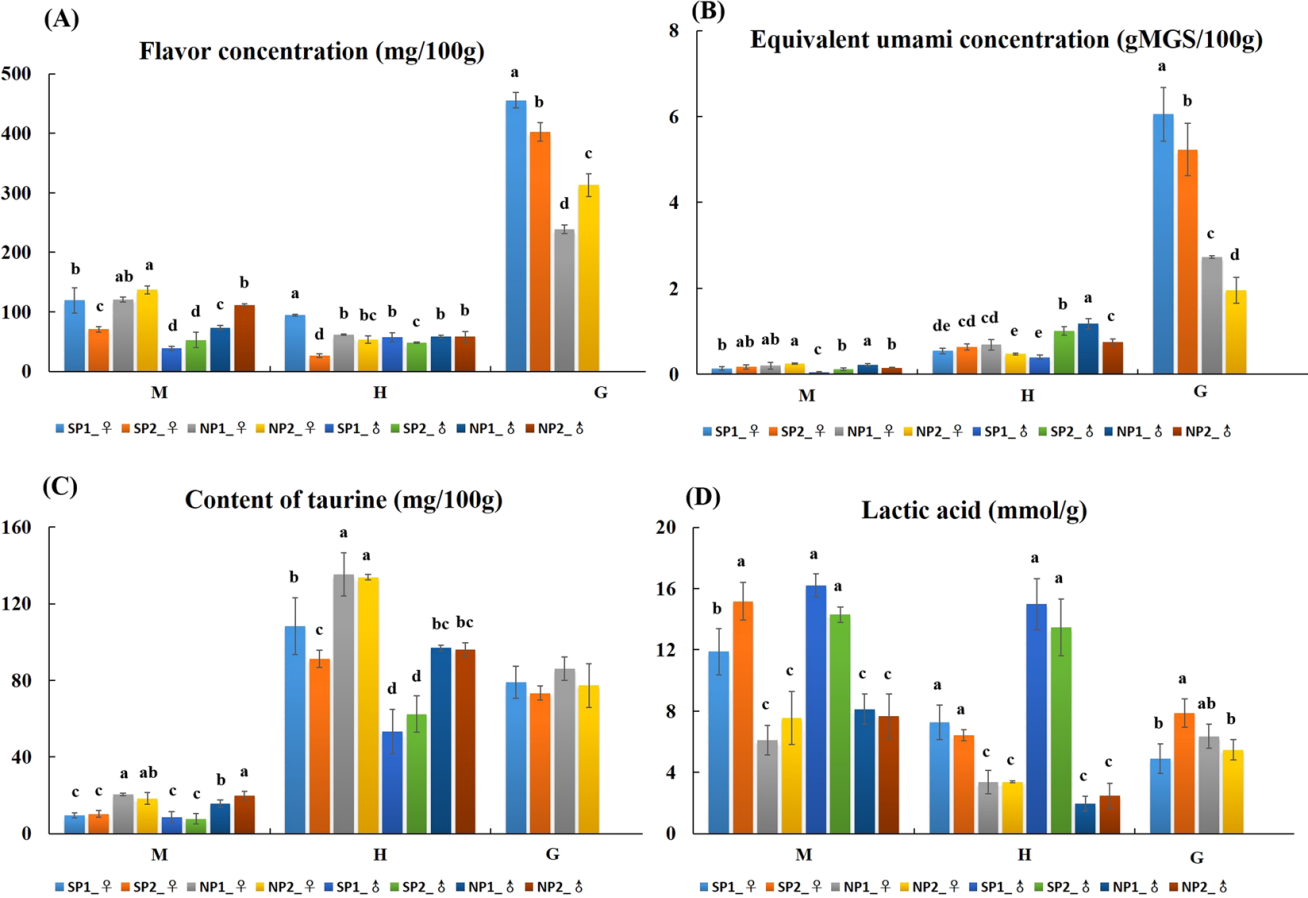
Content of flavor nucleotides (**A**), equivalent umami concentration (EUC) (**B**), Content of taurine (**C**) and lactic acid (**D**) in the edible parts of *S*. *paramamosain* from four areas. Flavor nucleotides included AMP, CMP, and IMP. EUC (g MSG/100 g) was the umami flavor intensity of a certain concentration of MSG equivalent to umami intensity was produced synergistically by the mixture of umami amino acids and flavor nucleotides^27^. Different letters above the columns indicate a significant differences (P < 0.05) between SP1-♀, SP2-♀, NP1-♀, NP2-♀, SP1-♂, SP2-♂, NP1-♂ and NP2-♂ respectively in muscle (M), hepatopancreas (H), and gonads (G).

Comparison between content of flavor nucleotides (Fig. 4A and Tables S2–4) of edible parts of SP and NP mud crabs showed that the highest content was in gonads, followed by muscles and hepatopancreas; but the intensity of umami flavor was strongest in gonads, followed by hepatopancreas and muscles (Fig. 4B). There were no significant differences in content of flavor nucleotides within each sex of crabs hepatopancreas and muscles, and content of flavor nucleotides and flavor intensity were highest in gonads compared with other edible parts; and SP-♀ was significantly higher than that of NP-♀ in content of flavor nucleotides, with SP1-♀ were the highest, 455.64 ± 12.99 mg/100 g and 7.05 ± 0.62 g MSG/100 g, respectively.

### Comparison of taurine and lactic acid between NP and SP crabs

Taurine content is highest in the hepatopancreas, followed by gonads, and lowest in muscles. (Fig. 4C). SP was significantly lower than NP in hepatopancreas and muscles except gonads. Therefore, the low temperature environment may be beneficial to taurine accumulation of *S*. *paramamosain*. The content of lactic acids in muscles of *S*. *paramamosain* was greater than in hepatopancreas and gonads, and the highest was in SP1-♂ of muscle (16.21 ± 0.74 mmol/g) (Fig. 4D). Lactic acids from SP in muscles and hepatopancreas were greater than that of corresponding NP values, and no differences was observed in gonads.

### Comparative analysis of gut microbiota diversity in SP and NP crabs

**Figure 5:**
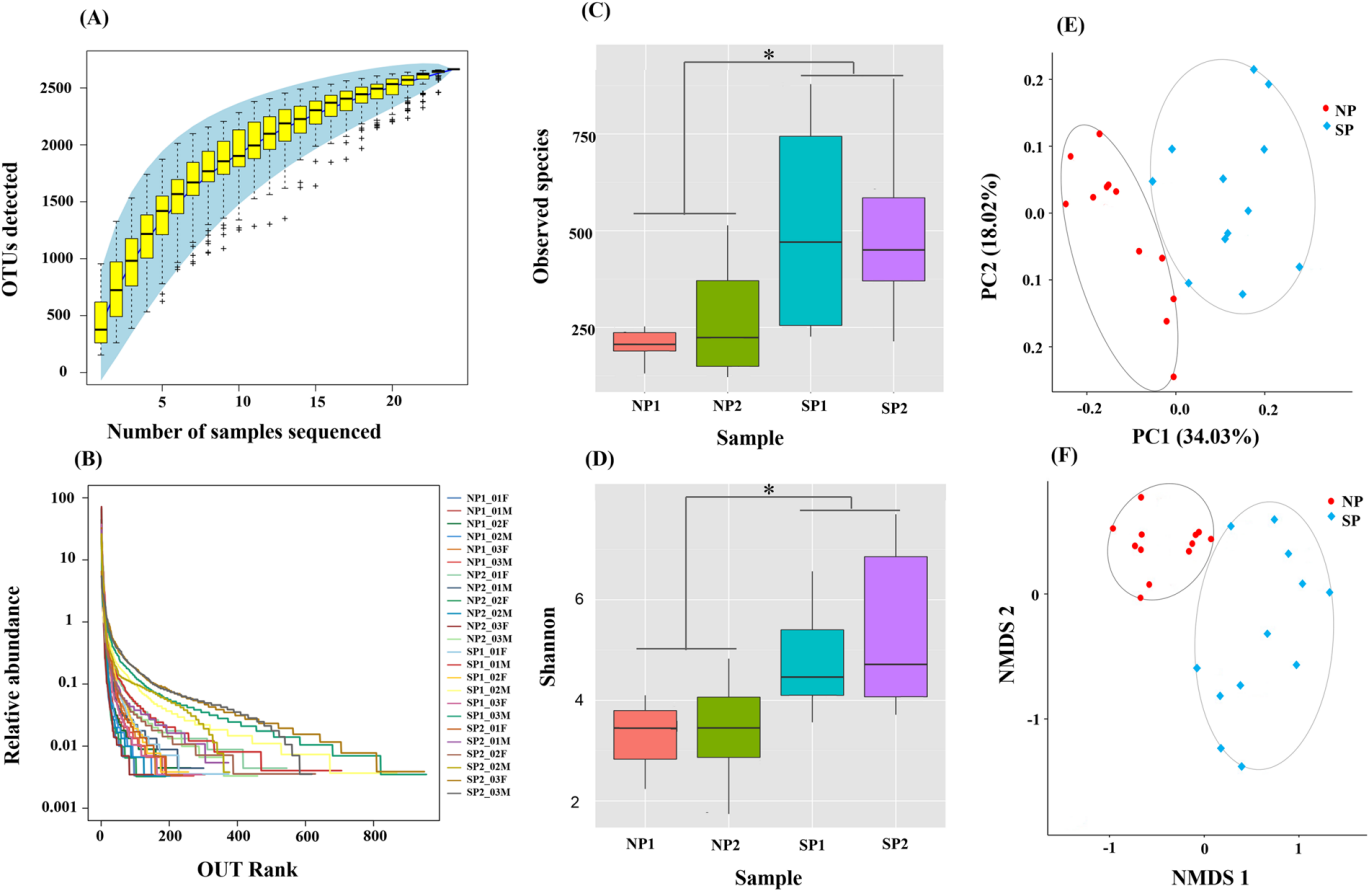
Species accumulation curves (**A**), rank abundance curves (**B**), alpha diversity analysis (Observed species (**C**) and Shannon index (**D**)) and beta diversity analysis (PCoA (**E**) and NMDS (**F**)) of gut microbiota community structures of SP and NP. In species accumulation curves (**A**), the x-axis indicates number of samples while yaxis shows the number of OTUs detected, If the curve shows a sharp rise, it indicates that with the increase of sampling quantity, a large number of new species will be discovered, that is, insufficient sampling quantity. If the curve flattens out, it means that species in this environment do not increase significantly with the increase of sample size, which indicates that sampling is sufficient. In rank abundance curves (**B**), the x-axis indicates the order number ranked by the OTUs abundance while yaxis shows the relative abundance of OTUs. The higher the richness of the species, the larger the span of the curve on the horizontal axis. In the vertical direction, the smoother the curve, the more uniform the species distribution. For PCoA (**E**) were analyzed with weighted Unifrac distance. and NMDS (**F**) were analyzed with Bray Curtis distance.

After high-throughput sequencing of all samples, the data sets were then subjected to quality control procedures which resulted in 655286 valid tags for the 24 samples analyzed. The data quantity of valid tags of each sample was between 24332 and 36438, the number of OTU is between 216 and 843, and the observed species was between 186 and 762 (Table 4). Alpha diversity indexes including Observed species, Shannon, Simpson, Chao1 and Good coverage were shown in Table 4. Specaccum species accumulation curve of alpha diversity analysis was used to estimate species abundance (Fig. 5A), the curve tends to flatten after 10, which indicates that the number of samples is sufficient and the results were reliable; Rank Abundance analysis evaluated the abundance and distribution of bacterial taxon (Fig. 5B), the curve was relatively smooth, and the OTU ranked in 100–600 species were evenly distributed, indicating that the species were evenly distributed.

**Table 4:** Overview of the sequencing data and alpha-diversity of the samples from the 4 regions samples in the *S*. *paramamosain*.

Samples	Valid tags	OTU counts	Observed species	shannon	simpson	chao1	Good coverage
NP1.F	27211 ± 4160	216 ± 49	186 ± 54	2.925 ± 0.873	0.718 ± 0.181	269.150 ± 64.163	0.996 ± 0.001
NP1.M	29611 ± 848	270 ± 36	219 ± 31	3.664 ± 0.383	0.868 ± 0.023	338.684 ± 53.187	0.995 ± 0.001
NP2.F	27430 ± 4268	296 ± 217	261 ± 219	3.113 ± 1.573	0.706 ± 0.227	360.292 ± 219.861	0.995 ± 0.003
NP2.M	27644 ± 4342	321 ± 130	283 ± 120	3.691 ± 0.510	0.840 ± 0.033	364.881 ± 123.618	0.995 ± 0.001
SP1.F	27659 ± 1374	309 ± 61	257 ± 44	3.922 ± 0.311	0.859 ± 0.034	406.785 ± 87.150	0.994 ± 0.001
SP1.M	26863 ± 1954	843 ± 126	762 ± 123	5.656 ± 0.877	0.918 ± 0.017	945.957 ± 54.250	0.988 ± 0.002
SP2.F	27679 ± 1767	606 ± 356	544 ± 339	5.196 ± 2.175	0.901 ± 0.076	695.696 ± 386.576	0.992 ± 0.005
SP2.M	24332 ± 5197	457 ± 141	447 ± 133	5.553 ± 1.703	0.913 ± 0.068	475.648 ± 136.186	0.997 ± 0.001

SP1 (Wangning county, Hainan province); SP2 (Yangjiang city, Guangdong province); NP1 (Sanmen county, zhejiang province); NP2 (Ninghai county, zhejiang province). F and M, indicate female crabs, male crabs.

The boxplots of alpha diversity were shown in Fig. 5C,D. Observed species and Shannon diversity indices were tested for the significance of discrepancies between the SP and NP two sample groups (P = 0.035 and 0.024, respectively), the results showed that the amount of OTU and abundance of bacteria in SP were significantly higher than that in NP, and the distribution of SP bacteria was more uniform than that in NP.

The principal co-ordinates analysis (PCoA) and nonmetric multidimensional scaling analysis (NMDS) of beta diversity were shown in Fig. 5E,F. Beta diversity analysis adopts weighted unifrac distance algorithm. According to the PCoA analysis, Samples of SP and NP are basically separated by 0.0 on the X-axis. Samples from the two regions were clustered together. According to the NMDS analysis, sample distribution of SP and NP can be well distinguished, while the sample distribution of SP was relatively loose. In conclusion, according to the results of alpha diversity analysis and Beta diversity analysis, the species abundance and diversity distribution of SP and NP samples are significantly different.

### Gut microbiota composition and relative abundance in SP and NP crabs

**Figure 6:**
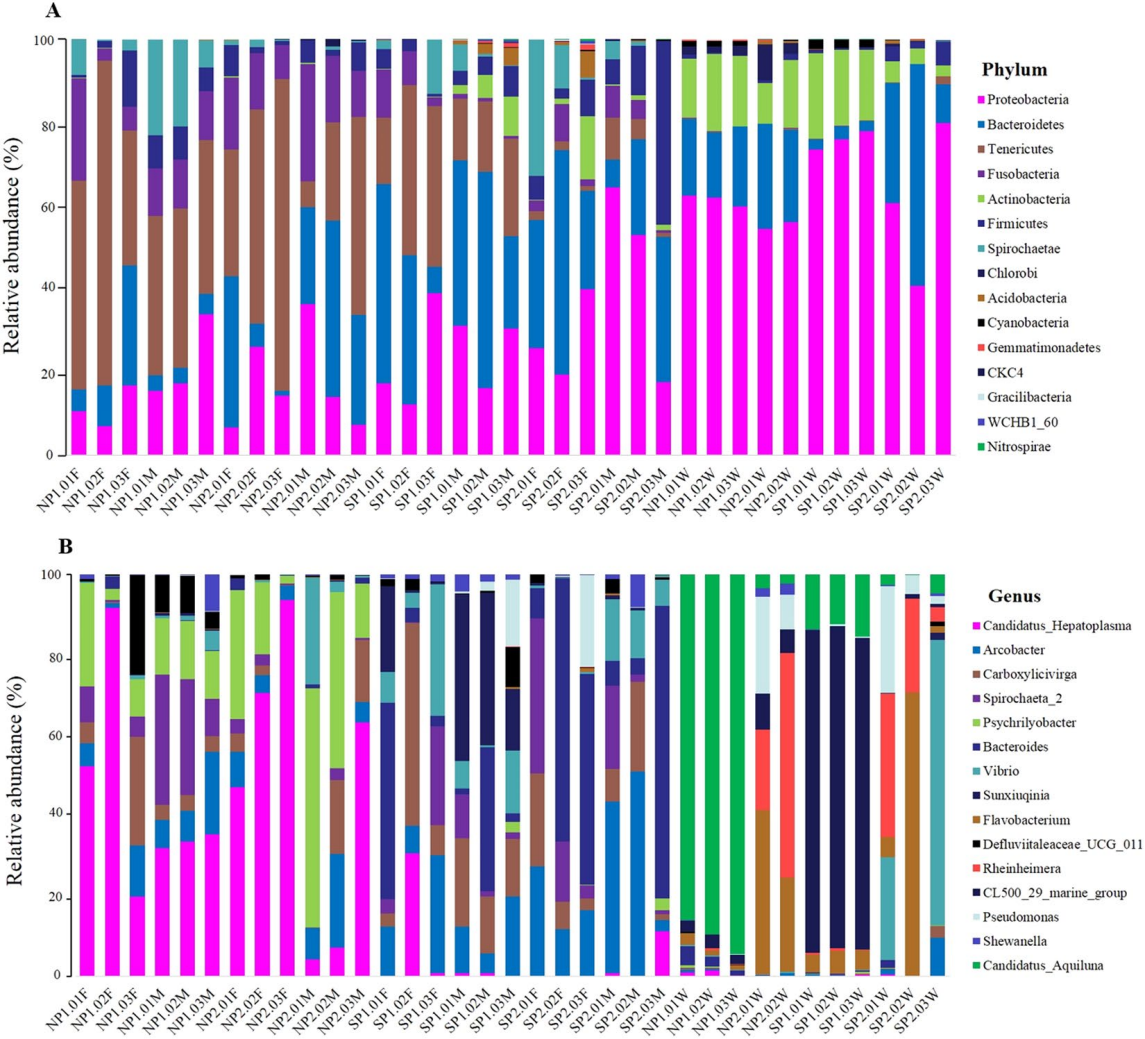
Comparison of the relative abundance of crab gut microbiota in four areas in the top 15 of phylum and genus levels. (**A**) Bar-plots showing the abundance distribution of the 15 most abundant phyla. (**B**) Bar-plots showing the abundance distribution of the 15 most abundant genera. The x-axis and y-axis represents the information of samples and relative abundance respectively.

Overall, we identified 30 phyla, 58 classes, 123 orders, 249 families and 481 genera of bacteria in the gut microbiota community from 24 gut of *S*. *paramamosain*. Comparison of the relative abundance of crab gut in four areas in the top 15 of phylum and genus levels was shown in Fig. 6. At the phylum level (Fig. 6A), Tenericutes was the most predominant phylum of NP, in which NP1.02 F (78.0%) reaches the highest relative abundance, while Tenericutes of SP have a low relative abundance and not dominant. Besides, Bacteroidetes, Proteobacteria and Fusobacteria were also dominant phylum of NP, contributed to the total composition. For SP, Bacteroidetes and Proteobacteria were dominant phyla with relative abundance higher than NP. At the genus level (Fig. 6B), *Candidatus Hepatoplasma* was the most predominant genus of NP, followed by *Psychrilyobacter*, and these two genera in only a small amount of samples in SP. For SP, the bacteria group was evenly distributed and no genus has an absolute dominant position. *Arcobacter*, *Bacteroides* and *Carboxylicivirga* contribute significantly to microbiota composition of SP, but the relative abundance of NP samples was very low. In conclusion, according to the relative abundance histogram results of gut microbiota composition at phylum and genus levels, there were significant differences in the distribution of NP and SP microbiota composition, as well as significant differences in the dominant microbiota composition of the two area. In addition, microbiota composition of SP has a high distribution richness and no absolute dominant bacteria, while NP has absolute dominant bacteria and its microbiota richness was lower than SP.

### Redundancy analysis

**Figure 7:**
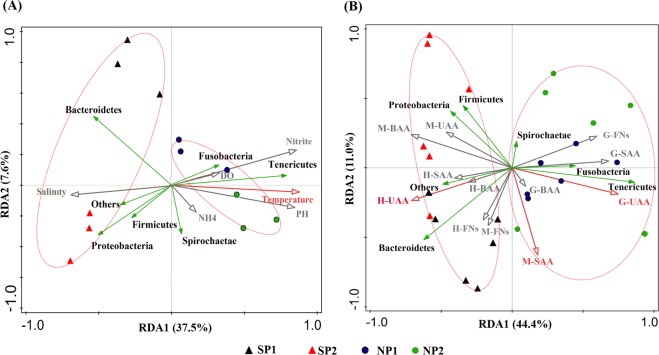
Redundancy analysis (RDA) was performed to explore the correlations between bacteria at the phylum level of percentage and envionment variables (**A**), flavor substances (**B**). RDA of phyla (green arrows) and tenvionment variables (**A**), flavor substances (**B**) (red arrow and gray arrow) in the Four sample collection points. The red arrow indicates a significant correlation with the bacteria (P < 0.05), while the gray indicates a non-significant correlation. Triangle, SP1 andSP2; dot, NP1 and NP2. (There were no gender differences in environmental variables in each region, and there were gender differences in flavor substances, so the coordinate points of the distribution of female samples and male samples in (**A**) were exactly the same).

Redundancy analysis (RDA) was performed to explore the correlations between gut microbiota at the phyla level of percentage and envionment variables (Fig. 7A), content of flavor substances (Fig. 7B), respectively. Samples from two collection points in southern region (SP) were distributed in the negative region to the left of RDA1, while samples from two collection points in northern region (NP) were distributed in the positive region to the right of RDA1(Fig. 7). The correlations analysis results of environmental variables and gut microbiota showed, RDA1 and RDA2 explained 37.5% and 7.6% of the total variance, The correlations between temperature and relative abundance of gut microbiota was significant (P = 0.02), while the other environmental variables were insignificant (Fig. 7A). Meanwhile, temperature also explains for 29.4% and contributing for 58.0% of the variable, respectively (Table S8). Temperature demonstrated positive correlations with the abundance of Fusobacteria and Tenericutes. Therefore, temperature is the most important variable affecting the distribution of gut microbiota diversity. The correlations analysis results of relative abundance of gut microbiota and flavor substances showed, RDA1 and RDA2 explained 44.4% and 11.0% of the total variance. The correlations between gut microbiote with content of G-UAA, H-UAA and M-SAA was significant, respectively (P = 0.002, 0.044, 0.038)). Meanwhile, the explains and contribution value of G-UAA was the largest, 25.6% and 38.7% respectively (Table S9). G-UAA demonstrated positive correlations with the abundance of Fusobacteria and Tenericutes, negative correlations with Firmicutes and Proteobacteria; H-UAA demonstrated positive correlations with the abundance of Bacteroidetes and M-SAA demonstrated negative correlations with Firmicutes and Proteobacteria. Therefore, there were a significant correlations between the diversity distribution of gut microbiota and the content of flavor substances.

## Discussion

The market price of mud crabs varies greatly due to the different flavor, with good quality mud crabs costing up to US $20/kg more than those of lower quality. Therefore, improving the quality of cultured mud crabs is of great significance for development of this industry. Many factors affect the quality of raised animals, including breeding areas, environment, and food^30,39,40–43^. In this study, mud crabs were raised in an ecotrophic culture mode in outside ponds. All conditions except temperature, including seedling density, feed rations, water exchange cycles, and pond environmental control were kept similar to ensure that temperature was the only factor affecting the quality and flavor of the crabs.

The main flavors of aquatic products include sweetness, umami, and bitterness. Organic acids such as lactic acid are very important to the taste of aquatic products^22,23,30,44^. The edible parts of crabs include muscles, hepatopancreas and gonads. Muscles has the highest levels of SAA, and shows obvious sweetness; gonads have high free nucleotide levels and EUC, and shows obvious umami; hepatopancreas has high levels of free BAAs, umami amino acids, and flavor nucleotides, and shows umami with bitterness flavors. The results were consistent with those of the *E*. *sinensis*^20,24,30,42^. By comparing the flavor substances of NP and SP crabs (Fig. 3), in general, the flavor amino acid content of NP was significantly higher than that of SP in muscles and hepatopancreas. There was no significant differences in the accumulation of flavor nucleotides (Fig. 4). In gonads, the flavor amino acids and nucleotides of SP were significantly higher than those of NP. Therefore, in this study, for SP and NP when the culture protocol and water parameters were identical, the temperature seems the possible reason of the different gonadal development, flavor substances. We suggest that higher temperature in southern areas may be beneficial to the accumulation of flavor substances in gonads, while lower temperature in northern areas may be beneficial to the accumulation of flavor substances in muscles and hepatopancreas. In addition, Studies have indicate that temperature may be the main factor for the differences of flavor substance content. Such as, the culture temperature was found to affect the development and biochemical composition of cancrid crab (*Cancer setosus* Molina)^45^, and the diel temperature fluctuation was reported to alter the nutrition of the sea cucumber (*Apostichopus japonicus* Selenka)^46^ and tongue sole (*Cynoglossus semilaevis*)^47^. The content of lactic acid and taurine was determined in this study. The results showed that the lactic acid content in edible parts of SP was significantly higher than that of NP, and the accumulation of lactic acid in muscles was the highest in both areas. This may have something to do with the amount of movement of the crab itself. The warmer the temperature, the more active the crab. Taurine mainly accumulates in the hepatopancreas, and the low temperature in NP promotes its accumulation.

Through high-throughput sequencing of 16S rRNA, we revealed the diversity distribution of gut microbiota of *S*. *paramamosain* in four areas of north and south China. The results showed that there were significant differences in the distribution of gut microbiota in NP and SP. Meanwhile,the gut microbiota richness of SP was higher than that of NP (Fig. 5). The dominant phylum of SP were Bacteroidetes and Proteobacteria respectively For NP, Tenericutes was the most predominant phylum, while Tenericutes of SP have a low relative abundance and not dominant. Besides, Bacteroidetes, Proteobacteria and Fusobacteria were also dominant phylum, contributed to the total composition (Fig. 6). Bacteroidetes, Proteobacteria, Tenericutes and Fusobacteria were several phylum with relatively high abundance of *S*. *paramamosain*, which is similar to the research results of *E*. *sinensis*^48–50^, and indicating that these bacteria were closely related to the growth, development and nutrition metabolism of *S*. *paramamosain*. These dominant bacteria may pose central roles in gut functions or were well adapted to the digestive tract^48,50^. The differences in the abundance of dominant bacteria in the gut of SP and NP crabs may be the potential factor leading to the differences in flavor.

The above results showed that SP and NP crabs were different in the diversity of bacteria and the accumulation of flavor substances. In order to reveal the relationship between environmental temperature and the diversity of intestinal flora and the content of flavor substances, RDA was performed to explore the correlations between relative abundance of gut microbiota at the phylum level with envionment variables and content of flavor substances (Fig. 7). The correlations between temperature and relative abundance of gut microbiota was significant (P = 0.02), while the other environmental variables were insignificant. Meanwhile, the correlations between gut microbiote with content of flavor substance was significant (G-UAA, H-UAA and M-SAA, respectively (P = 0.002, 0.044, 0.038). Thus, the temperature may be the main factors for the differences of flavor substances between SP and NP, which was most probably mediated by gut microbiota. But,whether the differences in gonadal development and flavor substances came for the absolute temperature values or/and the extent of the variation still needs further exploration. In addition, temperature demonstrated positive correlations with the abundance of Fusobacteria and Tenericutes. Fusobacteria and Tenericutes demonstrated positive correlations with content of flavor substances. Therefore, Fusobacteria and Tenericutes can be considered as important objects in the development of probiotics^48–50^.

In summary, in this study, the contents of flavor substances in *S*. *paramamosain* from four areas in the SP and the NP were systematically described and the differences of flavor substances and diversity of gut microbiota in SP and NP were compared. It is revealed that temperature potentially induced distinctive flavor of mud crab *S*. *paramamosain* mediated by gut microbiota. Meanwhile, the result of diversity distribution of gut microbiota will not only help researchers understand the assembly of the gut microbiota of *S*. *paramamosain*, but also provides reliable theoretical support for follow-up studies of host–microbiota relationships and the development of probiotics. It will also help to improve the precise management of crabs in aquaculture, and to promote the development of green crab breeding.

## Conclusions

In this study, we examined *S*. *paramamosain* collected within the main producing areas in China that were north sampling point (NP) and south sampling point (SP). Comparisons between flavor substances of edible parts showed that higher temperature in SP may be beneficial to the accumulation of flavor substances in gonads, while lower temperature in northern areas may be beneficial to the accumulation of flavor substances in muscles and hepatopancreas. The gut microbiota of crabs was analyzed via high-throughput sequencing results showed that there were significant differences in the distribution of gut microbiota in NP and SP. Meanwhile,the gut microbiota richness of SP was higher than that of NP.

The correlations between temperature and relative abundance of gut microbiota was significant. Meanwhile, the correlations between gut microbiote with content of flavor substance was significant. It is revealed that temperature may be the main factor for the difference of flavor substance content between SP and NP. Due to the difference in temperature, the diversity distribution of gut microbiota and dominant species of crabs are affected, thus affecting the quality and flavor of crabs, which may be the most likely way for temperature to affect the quality and flavor of crabs. Further exploration is needed with laboratory experiments in which the environment is more precisely controlled if these views are to be determined.

## Supplementary information

Supplementary information.

## Data Availability

Raw sequences were deposited into the NCBI Sequence Read Archive (SRA) database with accession numbers SRP215842.
