# Supplementary material for: Temperature potentially induced distinctive flavor of mud crab Scylla paramamosain mediated by gut microbiota
Source: Sci Rep. 2020 Feb 28;10:3720. doi: 10.1038/s41598-020-60685-0 (PMC7048764; doi:10.1038/s41598-020-60685-0)
Supplement: Supplementary file 1 — Supplementary information. [file 41598_2020_60685_MOESM1_ESM.docx]

# Temperature potentially induced distinctive flavor of mud crab *Scylla paramamosain* mediated by gut microbiota

Lei Tang^1^, Huan Wang^1, 2 #^, Chunlin Wang^1, 2^, Changkao Mu^1, 2^, Hongling Wei^1^, Hongzhi Yao^1^, Chunyu Ye^3, 4^, Lizhi Chen^3, 4^, Ce Shi^1, 2^

1 School of Marine Science, Ningbo University, Ningbo 315211, Zhejiang, China.

2 Key Laboratory of Applied Marine Biotechnology, Ministry of Education, Ningbo University, Ningbo 315211, Zhejiang, China.

3 Marine and Fishery Bureau, Sanmen County, Zhejiang Province

4 Fishery Technology Station, Sanmen County, Zhejiang Province

^🞸^ Corresponding author: wanghuan1@nub.edu.cn (Huan Wang)

Other authors: [791888694@qq.com](mailto:791888694@qq.com) (Lei Tang), wangchunlin@nbu.edu.cn (Chunlin Wang), muchangkao@nbu.edu.cn (Changkao Mu), [1357501364@qq.com](mailto:1357501364@qq.com) (Hongling Wei), 1025801555@qq.com (Hongzhi Yao), smhyj@163.com (Chunyu Ye), 80079745@qq.com (Lizhi Chen), shice3210@126.com (Ce Shi).

**Table S1** **Temperature, salinity, dissolved oxygen (DO), ammonia-nitrogen，nitrite and pH of northern sample point (NP) and southern sample point (SP).**

| **Month** | **Region** | **Temperature (℃)** | **Salinity** | **DO (mg/L)** | **Ammonia-nitrogen (mg/L)** | **Nitrite (mg/L)** | **pH** |
| --- | --- | --- | --- | --- | --- | --- | --- |
| **April** | SP1 | 26.02 ± 3.67a | 15.33 ± 1.98 | 8.98 ± 0.88 | 0.53 ± 0.11 | 0.12 ± 0.05 | 7.91 ± 0.23 |
|  | SP2 | 23.58 ± 3.56a | 16.12 ± 1.66 | 8.66 ± 0.92 | 0.54 ± 0.12 | 0.13 ± 0.06 | 8.01 ± 0.19 |
|  | NP1 | 18.22 ± 6.67b | 16.51 ± 1.55 | 8.24 ± 0.75 | 0.41 ± 0.12 | 0.13 ± 0.07 | 8.12 ± 0.26 |
|  | NP2 | 18.05 ± 6.57b | 17.11 ± 1.46 | 8.12 ± 0.67 | 0.42 ± 0.11 | 0.16 ± 0.05 | 8.56 ± 0.22 |
| **May** | SP1 | 28.1 ± 3.24a | 16.25 ± 2.32 | 8.52 ± 0.77 | 0.56 ± 0.09 | 0.12 ± 0.04 | 8.15 ± 0.27 |
|  | SP2 | 26.65 ± 3.26a | 15.14 ± 2.11 | 8.11 ± 0.86 | 0.66 ± 0.08 | 0.13 ± 0.04 | 8.16 ± 0.24 |
|  | NP1 | 22.68 ± 5.57b | 18.25 ± 1.81 | 8.01 ± 0.89 | 0.58 ± 0.09 | 0.12 ± 0.03 | 8.25 ± 0.1 |
|  | NP2 | 22.34 ± 5.49b | 17.11 ± 1.61 | 8.25 ± 0.84 | 0.53 ± 0.11 | 0.11 ± 0.05 | 8.11 ± 0.15 |
| **June** | SP1 | 29.8 ± 3.21a | 15.23 ± 2.38 | 7.56 ± 0.92 | 0.79 ± 0.13 | 0.13 ± 0.03 | 8.25 ± 0.14 |
|  | SP2 | 29.05 ± 3.26a | 14.02 ± 2.24 | 7.59 ± 0.91 | 0.78 ± 0.12 | 0.14 ± 0.04 | 8.14 ± 0.16 |
|  | NP1 | 25.13 ± 3.95b | 16.25 ± 1.98 | 7.66 ± 0.95 | 0.77 ± 0.11 | 0.13 ± 0.05 | 8.23 ± 0.11 |
|  | NP2 | 24.42 ± 3.98b | 17.01 ± 2.02 | 7.26 ± 0.87 | 0.76 ± 0.19 | 0.14 ± 0.06 | 8.23 ± 0.13 |
| **July** | SP1 | 31.44 ± 3.11 | 13.22 ± 2.13 | 5.26 ± 0.89 | 0.8 ± 0.08 | 0.13 ± 0.02 | 8.17 ± 0.24 |
|  | SP2 | 31.06 ± 3.23 | 13.56 ± 2.02 | 5.61 ± 0.88 | 0.75 ± 0.07 | 0.12 ± 0.03 | 8.13 ± 0.12 |
|  | NP1 | 29.82 ± 5.81 | 15.61 ± 1.88 | 5.26 ± 0.96 | 0.76 ± 0.08 | 0.12 ± 0.04 | 8.19 ± 0.15 |
|  | NP2 | 30.16 ± 5.86 | 15.81 ± 1.76 | 5.17 ± 0.87 | 0.79 ± 0.09 | 0.12 ± 0.05 | 8.05 ± 0.21 |
| **August** | SP1 | 31.47 ± 3.53 | 13.56 ± 1.99 | 6.22 ± 0.98 | 0.86 ± 0.07 | 0.14 ± 0.05 | 8.09 ± 0.21 |
|  | SP2 | 30.79 ± 3.61 | 13.55 ± 2.02 | 6.26 ± 0.99 | 0.85 ± 0.08 | 0.15 ± 0.04 | 8.11 ± 0.23 |
|  | NP1 | 30.34 ± 5.23 | 15.01 ± 1.53 | 6.15 ± 0.86 | 0.88 ± 0.08 | 0.14 ± 0.03 | 8.12 ± 0.19 |
|  | NP2 | 30.23 ± 5.46 | 14.25 ± 1.43 | 6.11 ± 1.01 | 0.91 ± 0.11 | 0.17 ± 0.06 | 8.13 ± 0.17 |
| **September** | SP1 | 28.93 ± 3.51a | 14.11 ± 2.01 | 6.55 ± 1.25 | 0.95 ± 0.11 | 0.15 ± 0.04 | 8.06 ± 0.18 |
|  | SP2 | 29.45 ± 3.75a | 13.86 ± 1.96 | 6.88 ± 0.98 | 0.96 ± 0.13 | 0.17 ± 0.01 | 8.04 ± 0.23 |
|  | NP1 | 26.22 ± 4.86b | 15.12 ± 1.63 | 7.01 ± 0.87 | 0.93 ± 0.12 | 0.16 ± 0.03 | 8.05 ± 0.24 |
|  | NP2 | 25.52 ± 4.91b | 15.22 ± 1.69 | 7.12 ± 0.88 | 0.94 ± 0.08 | 0.15 ± 0.05 | 8.06 ± 0.18 |
| **October** | SP1 | 26.21 ± 3.34a | 13.25 ± 1.86 | 6.23 ± 0.96 | 0.99 ± 0.09 | 0.16 ± 0.06 | 8.04 ± 0.19 |
|  | SP2 | 25.34 ± 2.45a | 13.56 ± 1.43 | 6.57 ± 0.93 | 0.97 ± 0.08 | 0.15 ± 0.04 | 8.13 ± 0.17 |
|  | NP1 | 20.71 ± 5.32b | 14.53 ± 1.43 | 7.52 ± 0.97 | 1.01 ± 0.07 | 0.16 ± 0.03 | 8.23 ± 0.16 |
|  | NP2 | 20.35 ± 5.43b | 15.12 ± 1.89 | 7.11 ± 0.95 | 0.96 ± 0.11 | 0.16 ± 0.04 | 8.22 ± 0.19 |
| **November** | SP1 | 23.87 ± 2.82a | 13.25 ± 2.11 | 6.01 ± 0.94 | 0.96 ± 0.12 | 0.16 ± 0.05 | 8.26 ± 0.19 |
|  | SP2 | 21.30 ± 2.93a | 12.78 ± 2.01 | 6.12 ± 0.99 | 0.93 ± 0.10 | 0.15 ± 0.04 | 8.16 ± 0.16 |
|  | NP1 | 14.77 ± 5.17b | 14.23 ± 1.67 | 5.84 ± 0.93 | 0.95 ± 0.13 | 0.14 ± 0.03 | 8.17 ± 0.23 |
|  | NP2 | 14.53 ± 5.56b | 14..35 ± 1.77 | 5.87 ± 0.79 | 0.97 ± 0.09 | 0.13 ± 0.03 | 8.15 ± 0.21 |

**Table S2- Content of free nucleotides in muscle of *S. paramamosain* from different areas (mg/100g).**

| **Free nucleotides** | **females** | | | | **males** | | | |
| --- | --- | --- | --- | --- | --- | --- | --- | --- |
|  | **SP1-♀** | **SP2-♀** | **NP1-♀** | **NP2-♀** | **SP1-♂** | **SP2-♂** | **NP1-♂** | **NP2-♂** |
| **AMP** | 13.18 ± 1.78b | 0.51 ± 0.42c | 16.21 ± 1.18b | 15.37 ± 1.90b | 14.48 ± 5.88b | 1.07 ± 0.14c | 12.6 ± 3.08b | 23.88 ± 2.72a |
| **GMP** | 96.89 ± 19.23a | 55.48 ± 3.92b | 96.11 ± 3.53a | 105.52 ± 5.45a | 17.53 ± 2.20c | 44.5 ± 12.41b | 49.89 ± 4.67b | 58.29 ± 1.70b |
| **IMP** | 5.53 ± 0.38cd | 3.29 ± 0.83de | 6.03 ± 0.51c | 9.01 ± 2.19b | 5.07 ± 0.50cde | 3.09 ± 1.56e | 3.84 ± 1.90cde | 25.94 ± 0.73a |
| **CMP** | 0.72 ± 0.10de | 2.91 ± 0.57a | 0.99 ± 0.06cd | 0.39 ± 0.13ef | 0.61 ± 0.09de | 1.32 ± 0.09c | 0.11 ± 0.01f | 1.72 ± 0.16b |
| **UMP** | 2.93 ± 0.38b | 8.74 ± 2.02a | 1.75 ± 0.18bc | 6.81 ± 0.86a | 0.70 ± 0.18c | 2.78 ± 0.22bc | 7.13 ± 2.25a | 1.39 ± 0.29bc |
| **Total nucleotides** | 119.25 ± 21.41b | 70.93 ± 4.67c | 121.09 ± 4.21ab | 137.1 ± 6.87a | 38.39 ± 3.91d | 52.76 ± 13.31d | 73.58 ± 3.85c | 111.22 ± 1.89b |

In the same row, different letters means significant difference; (P < 0.05). SP1 (Wangning county, Hainan province); SP2 (Yangjiang city, Guangdong province); NP1 (Sanmen county, zhejiang province); NP2 (Ninghai county, zhejiang province). -, indicate could not be determined.

**Table S3 - Content of free nucleotides in hepatopancreas of *S. paramamosain* from different areas (mg/100g).**

| **Free nucleotides** | **females** | | | | **males** | | | |
| --- | --- | --- | --- | --- | --- | --- | --- | --- |
|  | **SP1-♀** | **SP2-♀** | **NP1-♀** | **NP2-♀** | **SP1-♂** | **SP2-♂** | **NP1-♂** | **NP2-♂** |
| **AMP** | 28.80 ± 6.02a | 0.36 ± 0.21e | 22.04 ± 0.97b | 12.12 ± 1.68c | 1.87 ± 0.62e | 0.35 ± 0.01e | 7.13 ± 0.66d | 16.10 ± 4.47c |
| **GMP** | 23.91 ± 4.75a | 4.42 ± 1.37b | 6.57 ± 2.89b | 8.62 ± 1.29b | 4.79 ± 1.78b | 6.28 ± 0.65b | 5.53 ± 2.50b | 5.60 ± 0.42b |
| **IMP** | 28.08 ± 3.14b | 8.81 ± 4.15e | 13.37 ± 0.51de | 13.27 ± 1.49de | 39.14 ± 8.9a | 24.82 ± 1.87bc | 21.51 ± 1.75bc | 19.09 ± 2.38cd |
| **CMP** | 1.99 ± 0.38ef | 12.47 ± 1.22b | 6.11 ± 1.35c | 0.15 ± 0.07f | 2.88 ± 1.02de | 16.87 ± 2.52a | 14.25 ± 1.94b | 4.78 ± 0.56cd |
| **UMP** | 11.82 ± 0.65bc | 0.00 ± 0.00e | 13.72 ± 1.92b | 19.11 ± 2.60a | 8.75 ± 1.61d | 0.00 ± 0.00e | 9.67 ± 0.89cd | 12.69 ± 1.83b |
| **Total nucleotides** | 94.6 ± 1.49a | 26.06 ± 3.11d | 61.82 ± 0.68b | 53.26 ± 6.28bc | 57.43 ± 7.70b | 48.32 ± 1.14c | 58.07 ± 2.40b | 58.26 ± 9.07b |

In the same row, different letters means significant difference; (P < 0.05). SP1 (Wangning county, Hainan province); SP2 (Yangjiang city, Guangdong province); NP1 (Sanmen county, zhejiang province); NP2 (Ninghai county, zhejiang province). -, indicate could not be determined.

**Table S4 - Content of free nucleotides in gonad of *S. paramamosain* from different areas (mg/100g).**

| **Free nucleotides** | **females** | | | |
| --- | --- | --- | --- | --- |
|  | **SP1-♀** | **SP2-♀** | **NP1-♀** | **NP2-♀** |
| **AMP** | 23.01 ± 4.84ab | 13.73 ± 1.78c | 25.72 ± 2.08a | 18.25 ± 4.3bc |
| **GMP** | 264.09 ± 8.34a | 234.28 ± 10.45b | 57.36 ± 11.03d | 197.67 ± 5.69c |
| **IMP** | 54.62 ± 7.77b | 49.74 ± 1.49b | 74.53 ± 5.83a | 56.34 ± 5.68b |
| **CMP** | 113.83 ± 13.14a | 60.05 ± 4.29bc | 81.26 ± 15.58b | 39.72 ± 14.41c |
| **UMP** | 0.09 ± 0.15b | 44.47 ± 5.20a | 0.00 ± 0.00b | 1.04 ± 0.97b |
| **Total nucleotides** | 455.64 ± 12.99a | 402.26 ± 15.42b | 238.87 ± 7.22d | 313.02 ± 19.39c |

In the same row, different letters means significant difference; (P < 0.05). SP1 (Wangning county, Hainan province); SP2 (Yangjiang city, Guangdong province); NP1 (Sanmen county, zhejiang province); NP2 (Ninghai county, zhejiang province). -, indicate could not be determined.

**Table S5- Equivalent umami concentration in edible parts of *S. paramamosain* from different areas (gMSG/100g).**

| **Tissue** | **EUC** | | | |
| --- | --- | --- | --- | --- |
|  | **SP1** | **SP2** | **NP1** | **NP2** |
| **M-♀** | 0.13 ± 0.05b | 0.17 ± 0.05ab | 0.20 ± 0.08ab | 0.25 ± 0.02a |
| **M-♂** | 0.05 ± 0.01b | 0.11 ± 0.03b | 0.22 ± 0.03a | 0.15 ± 0.01a |
| **H-♀** | 0.54 ± 0.07ab | 0.63 ± 0.07a | 0.69 ± 0.12a | 0.47 ± 0.03b |
| **H-♂** | 0.40 ± 0.05d | 1.00 ± 0.10b | 1.18 ± 0.12a | 0.74 ± 0.08c |
| **G-♀** | 7.05 ± 0.62a | 5.23 ± 0.61b | 2.73 ± 0.03c | 1.75 ± 0.55c |

In the same row, different letters means significant difference; (P < 0.05). SP1 (Wangning county, Hainan province); SP2 (Yangjiang city, Guangdong province); NP1 (Sanmen county, zhejiang province); NP2 (Ninghai county, zhejiang province). M, H, and G indicate muscle, hepatopancreas, and gonads respectively of S. paramamosain from different areas; ♀, females; ♂,males. -, indicate could not be determined.

**Table S6- Content of taurine in edible parts of *S. paramamosain* from different areas (mg/100g).**

| **Tissue** | **Content of taurine** | | | |
| --- | --- | --- | --- | --- |
|  | **SP1** | **SP2** | **NP1** | **NP2** |
| **M-♀** | 9.71 ± 1.27c | 10.28 ± 1.75c | 20.52 ± 0.63a | 18.38 ± 3.00a |
| **M-♂** | 8.59 ± 2.81c | 7.78 ± 2.72c | 15.71 ± 1.97b | 19.89 ± 2.32a |
| **H-♀** | 108.3 ± 14.82b | 91.27 ± 4.64c | 135.38 ± 11.23a | 133.84 ± 1.45a |
| **H-♂** | 53.34 ± 11.56b | 62.36 ± 9.47b | 96.99 ± 1.45a | 96.16 ± 3.61a |
| **G-♀** | 78.98 ± 8.29 | 73.39 ± 3.68 | 86.13 ± 6.01 | 77.32 ± 11.49 |

In the same row, different letters means significant difference; (P < 0.05). SP1 (Wangning county, Hainan province); SP2 (Yangjiang city, Guangdong province); NP1 (Sanmen county, zhejiang province); NP2 (Ninghai county, zhejiang province). M, H, and G indicate muscle, hepatopancreas, and gonads respectively of *S. paramamosain* from different areas; ♀, females; ♂,males. -, indicate could not be determined.

**Table S7-** **Content of lactic acid in edible parts of *S. paramamosain* from different areas (mmol/g).**

| **Tissue** | **Content of lactic acid** | | | |
| --- | --- | --- | --- | --- |
|  | **SP1** | **SP2** | **NP1** | **NP2** |
| **M-♀** | 11.88 ± 1.51b | 15.17 ± 1.23a | 6.11 ± 0.98c | 7.56 ± 1.73c |
| **M-♂** | 16.21 ± 0.74a | 14.3 ± 0.50a | 8.13 ± 0.98c | 7.66 ± 1.48c |
| **H-♀** | 7.27 ± 1.12a | 6.42 ± 0.37a | 3.36 ± 0.77b | 3.38 ± 0.06b |
| **H-♂** | 14.98 ± 1.68a | 13.47 ± 1.85a | 1.97 ± 0.49b | 2.47 ± 0.82b |
| **G-♀** | 4.90 ± 0.96b | 7.87 ± 0.94a | 6.37 ± 0.78ab | 5.47 ± 0.66b |

In the same row, different letters means significant difference; (P < 0.05). SP1 (Wangning county, Hainan province); SP2 (Yangjiang city, Guangdong province); NP1 (Sanmen county, zhejiang province); NP2 (Ninghai county, zhejiang province). M, H, and G indicate muscle, hepatopancreas, and gonads respectively of *S. paramamosain* from different areas; ♀, females; ♂,males. -, indicate could not be determined.

**Table S8- Data of correlations** **RDA analysis of** **envionment variables and gut microbiota.**

| **envionment variables** | **Explains (%)** | **Contribution (%)** | **P** |
| --- | --- | --- | --- |
| Temperature | 29.4 | 58.0 | 0.002* |
| pH | 5.2 | 10.2 | 0.16 |
| nitrite | 6.2 | 12.3 | 0.088 |
| ammonia nitrogen (AN) | 4.2 | 8.3 | 0.216 |
| Dissolved Oxygen (DO) | 3.5 | 6.9 | 0.292 |
| salinity | 2.2 | 4.4 | 0.548 |

**Table S9- Data of correlations RDA analysis of** **flavor substances and gut microbiota.**

| **flavor substances** | **Explains (%)** | **Contribution (%)** | **P** |
| --- | --- | --- | --- |
| G-UAA | 25.6 | 38.7 | 0.002* |
| H-UAA | 7.7 | 11.7 | 0.044* |
| M-SAA | 7.5 | 11.4 | 0.038* |
| G-SAA | 4.3 | 6.4 | 0.216 |
| H-BAA | 3.8 | 5.8 | 0.262 |
| M-UAA | 3.9 | 6 | 0.234 |
| H-Flavor nucleotide (H-FN) | 3.7 | 5.6 | 0.258 |
| M-Flavor nucleotide (M-FN) | 1.7 | 2.6 | 0.646 |
| H-SAA | 2.7 | 4.1 | 0.462 |
| G-BAA | 2.7 | 4.2 | 0.402 |
| G-Flavor nucleotide (G-FN) | 1.8 | 2.8 | 0.56 |
| M-BAA | 0.5 | 0.8 | 0.946 |

**
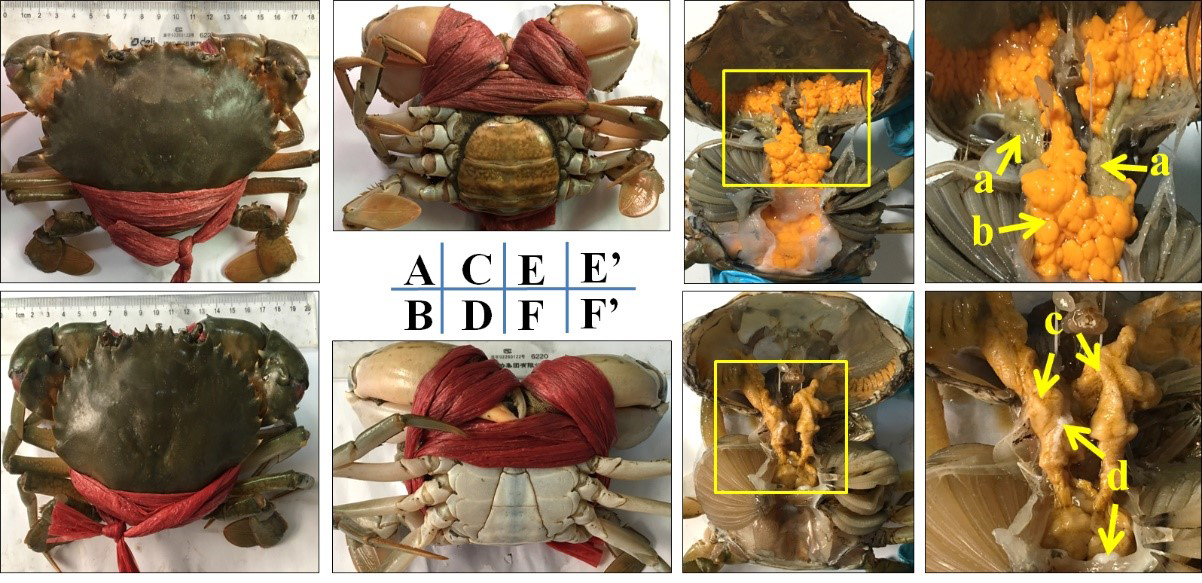
**

**Fig S1 Edible parts of *S. paramamosain*:** A, C, E, and E’ show females; B, D, F, and F‘ show males; E’ and F‘ are magnifications of highlighted boxes in E and F, respectively. (a) Hepatopancreas of female; (b) gonad of female; (c) hepatopancreas of male, pale white; and (d) gonad of male. Male and female individuals were from the southernmost point of the main producing area in China; mass (♀) 305±25g and (♂) 296±21g.


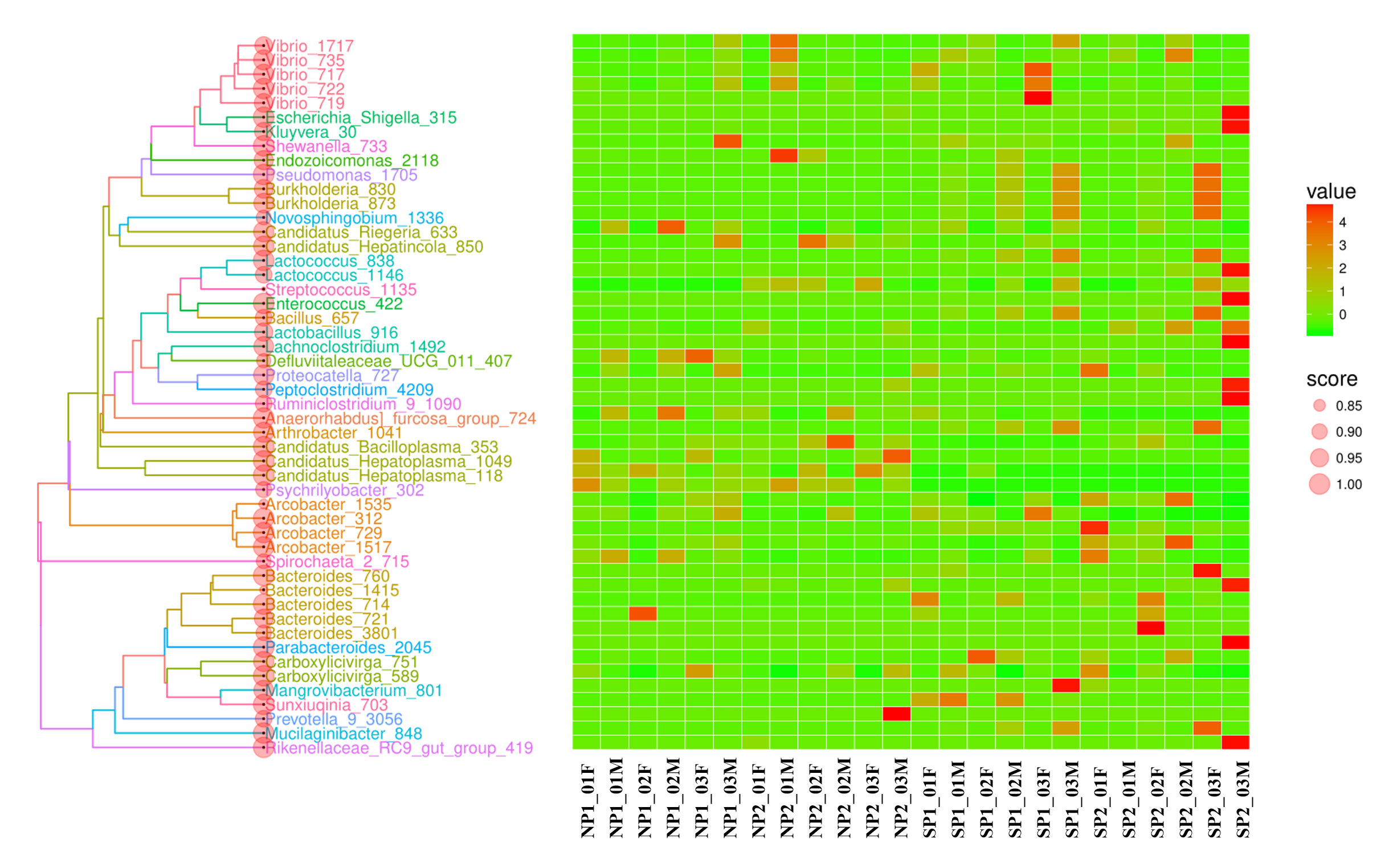


**Fig S2 Phylogenetic tree and OTU abundance map of TOP50 species of green crab.** On the left is the evolutionary tree graph, score value represents the credibility of evolutionary branches, and the annotation information is OTU generic horizontal annotation information plus the id number of OTU;The right is the abundance graph, corresponding to the abundance of OTU on the left in each sample.
